# Tri-State Medical Society of Alabama, Georgia and Tennessee—Ninth Annual Meeting

**Published:** 1897-12

**Authors:** 


### SOCIETY REPORTS.

### TRI-STATE MEDICAL SOCIETY OF ALABAMA, GEORGIA AND TENNESSEE—NINTH ANNUAL MEETING.

NASHVILLE, TENN., October 12, 13, 14.

The President, Dr. W. F. Westmoreland of Atlanta, called the meeting to order, and it was opened with prayer by Rev. J. B. Hawthorne.

Gov. Robert L. Taylor welcomed the Society to Nashville in an earnest and pleasing address. The President responded fittingly on behalf of the Society.

The President then delivered his annual address, "Carcinoma of the Breast," in which he deplored the fact that cases are referred to the surgeon too late for operation. He called especial attention to the importance of early diagnosis. Every tumor of the breast is suspicious. All writers agree that inflammation of the breast predisposes to cancer. Traumatism plays an important part in causation. In his experience, when there is a bad family history, the tumor will return. He called particular attention to Halsted's operation. The greatest infiltration is in the skin, next the subpectoral and axillary glands. Cells may be widely scattered early. Carcinoma extends through the vessels. The pectoralis minor should be left until the pectoralis major is excised. Everything that looks suspicious in the axilla should be removed. Nerves should be cut, especially if connected with infiltrated glands. Operation should be complete, even to excising of axillary veins. In a case where a complete operation did not seem necessary, there was a return in four months. If half can be saved it will be as good as can be expected. No living man can lose only six per cent. if the three-year rule is observed. Figures are fallacious. The large area, if left to granulate and cicatrize, predisposes to carcinoma. As a rule he grafts in a week. All cancer patients have a lack of red blood-corpuscles, the hemoglobin reduced to ninety per cent. When the hemoglobin is reduced fifteen per cent. patient will die. This accounts for many lost after operation with no apparent cause.

Dr. B. Sherwood Dunn, of Paris, France, said that he fully concurred with the position of the essayist, that the operation ought to remove not only such tissue as gave rise to suspicion, but to go further and remove tissue which he was satisfied to be healthy, for the reason that the line of demarcation cannot be readily defined. He felt that clinical experience did not support the favorable statistics reported by most operators, and that operations for carcinoma rarely resulted in permanent cure.

Dr. Paul F. Eve said that if all cancer cells were removed there would be no recurrence. He removes tissue which seems normal. Before regional infiltration cancer can be removed. Then there will be no return. We are looking to the day when the surgeon will recognize all cancer tissue and remove same.

In closing the discussion Dr. Westmoreland said that he would not try to cover wound by stretching skin over it. This is bad practice. In the last year had a large per cent. of cases in negroes, so that Dr. Cunningham's idea, which is also in Warren's Pathology, is not correct. The axilla cannot be cleaned out without removing pectoralis muscle and cutting veins which come from axillary plexus. Drawing up skin will restrict movements of arm.

Dr. Seale Harris, of Union Springs, Ala., read a paper, "The Arrest of Smallpox in the Vesicular Stage," based on the idea that smallpox would stop after the vesicular stage if there was no mixed infection, the idea being to prevent by applying antiseptics to the skin. Quoting from Bryan and Bibb, he related cases. In a case seen by the writer the treatment was successful. It prevents pitting, secondary fever, the intolerable itching and fearful stench of the pustular stage.

Dr. Llewellyn P. Barbour, Tullahoma, Tenn., read a paper on "The Pathology and Diagnosis of Early Phthisis," including in this term to the stage where there is breaking down of tissue. In some the diagnosis is easy, in others can be made only by investigating symptoms that may seem unimportant. Skill is required to appreciate physical signs, but the clinical symptoms will often make the diagnosis. Bacilli may not be found in undoubted case. Hemoptysis almost certainly due to tuberculosis. Pleuritic pains are characteristic of phthisis. May be absent, as may also increase of temperature. Absence of temperature indicates slow advancement or arrest. Take temperature every two hours, ten to twelve days. If increase two degrees with exercise, probably phthisis.

Dr. Paul Paquin, of St. Louis, said that the basis of tuberculosis was a battle between the tubercle bacilli and the cells in which they are lodged. The disease may exist a long time before the bacilli are expelled, hence importance of early diagnosis. This is not so difficult. Early clearing of throat important. An important factor is serous pneumonia. Temperature important, rarely above 101.

Dr. R. M. Cunningham insisted on importance of early diagnosis, which may be made from clinical signs alone. Bacilli may be in sputum of healthy. Altered ratio between pulse and temperature important. Pulse faster than for temperature. Physical signs show lesions, which may not be tubercular. There may be a tubercular nidus with no physical signs. The crepitant râle in apex, unilateral, is diagnostic of tuberculosis.

Dr. Andrew Boyd thought that enough attention was not paid to the minute clinical history; that this is lost sight of by going off to the unknown too much. The microscope is the *sine qua non*, but the subjective symptoms come first, hemoptysis, the pulse (out of all proportion to the temperature), the general malaise, dyspnea, loss of appetite, etc. These come first, then the physical signs, crepitant râle in apex, followed by active physical signs. To sum up, he thought the subjective signs are our first guide, then objective; the physical signs and microscope to dispel all doubts.

Dr. Paul Paquin, St. Louis, Mo., read a paper on "Sero-Therapy in Tuberculosis." The use of serums, nature's remedies, seems to promise much, as all other remedies have failed. Tuberculosis generally a mixed infection; with bacilli we find other germs. The nature of the mixed infection should be determined by the microscope. This is as important as the use of the microscope in the early stage. There is no one treatment applicable to all cases. Treatment should be combined. In mixed infection the serum should be prepared to meet the case. Hygienic measures should be employed, climate in certain cases—altitude not of much importance, the benefit being due to presence of ozone. Climate often tried too late, or may remain too short a time. Hydrotherapy often beneficial by stimulating circulation, also massage. In dieting, animal food best. Cream better than cod-liver oil; whisky and tobacco contraindicated.

Dr. J. A. Witherspoon said that this is an infectious disease, but does not have a period of invasion, no incubation; if self-limited, the limit is the grave. Would not say that none ever got well, but would say that none got well except by the inherent power of resistance. Has absolutely no confidence in serum treatment of tuberculosis. These gentlemen got good results, first, from the fact that some of the cases were not tuberculosis; second, to the hygienic and other measures are due the cure. The bacillus may be found without tuberculosis.

Dr. W. F. Westmoreland said that the idea that tuberculosis could not be cured should not be allowed to go unchallenged. Wherever the diseased area could be removed the disease could be cured. In view of the statistics, there can be no doubt of the good results from these serums.

Dr. Paquin said that he had been misunderstood. He had not used the word cure. Had reported the cases as he had found them. Tuberculin is not a serum, but the opposite. Tuberculosis and diphtheria are not biologically similar, and may require different treatment. His cases were not under favorable surroundings, were not picked. The methods were open, any physician could visit the laboratory.

Dr. Hazle Padgett, Columbia, Tenn., read a paper on "The Causes, Diagnosis and Treatment of Valvular Disease." As causes he mentioned especially acute rheumatism, Bright's disease, violent muscular effort. Diagnosis by timing murmurs, systolic, diastolic and presystolic. Obscure murmurs may be determined in horizontal position. Important in life insurance. Exercise may bring out murmur, also forced inspiration, then forced expiration, then suspend respiration. Future of patient should not be darkened by telling him he has heart disease. Tell some one who is interested in patient. Many will live years. Aortic stenosis most dangerous, mitral stenosis akin to it.

Dr. R. M. Cunningham, Birmingham, Ala., read a paper on "The Relation of the Cause to the Immediate and Remote Results and Associated Lesions of Fractures," in which he gave a study of fracture, based on mechanical principles, showing that bending, compression and torsion are the usual means of fracture. Lesions in bones, in surrounding tissue, in viscera. In indirect violence injury to soft parts less than in direct. We are sometimes deceived in the amount of violence and consequent injury, and prognosis should always be guarded.

Dr. Geo. S. Brown said that the amount of violence is the greatest factor in the prognosis, but when it is less than enough to cause total destruction of the circulation, it is only so as the cause of the infection, which is itself the most important determining factor.

Mr. Lister, walking his wards with his class twenty years ago, said: "Gentlemen, I have often noticed that bad contusions, not complicated by lesions of the skin, nearly always do well; those with lesions of the skin have trouble. I am not able to explain this, but the one who does will make his name immortal." He explained afterward himself, and his name is immortal.

Dr. J. B. Murfree said: "In the treatment of fractures the cause of the injury is too little considered. This must necessarily play an important part in the treatment. A fracture produced by great external violence must be treated with great care, and be so dressed as to be inspected, while one produced by muscular contraction may be put up in plaster of Paris and never inspected until the fracture has united.

"As stated, a simple fracture, as a rule, will do better than a compound one, but in every fracture, whether simple, compound or comminuted, we must duly consider the force that breaks the bone. I remember a case of fracture of the leg, produced by external violence, that was bandaged and splinted. The limb swelled, and the pressure of the bandage being painful, the bandage was cut. The leg mortified and had to be amputated, and the doctor was sued for malpractice. We must duly consider the damage done to the soft parts as well as the bone. A case came under my charge where a leg was mashed by a car. An eminent surgeon amputated at the knee-joint, but when the first dressing was removed the soft tissues were found to be mortified, and in time all the flaps sloughed, leaving the condyles of the femur projecting beyond the soft parts to be covered by granulation after months of suffering. So we must duly consider the injury done the soft parts from the cause producing the fracture. The force that breaks the bone and its mode of application must be considered in the treatment of every fracture."

Dr. J. M. Mathews, Louisville, Ky., addressed the society on "Cancer of the Rectum." He said in part that while extirpation was the proper treatment, still the application was limited. Authors claim that cases live four, five and seven years; he has a case in Louisville with cancer eight years. The rectum is divided into lower, upper and middle; lower one and a half inch. This may be removed. In middle third will surely return. Upper third involves sigmoid flexure. This may be cut out and anastomosis made; but why? Would limit operation to lower one and a half inch. Pain not always present. Doubt if operation prolongs life. Cancer patients live five to eight years. There is an allied condition which can be diagnosed only by experts of years' experience. The only treatment in inoperable cancer is to let patient down easily with opium.

Dr. George S. Brown of Birmingham, Ala., read a paper on "Metatarsalgia or Morton's Painful Toe." This is an affection of the fourth metatarsal phalangeal joint. The pain is sometimes beyond comprehension. Morton suggested excision of the joint. One symptom is necessity of removing shoe. Ligaments so stretched as to allow the bone to press against nerves. Thinks thin-soled shoe may be a factor in causation. Mild cases may be relieved by stiff sole. In severe cases the joint should be excised.

Dr. J. A. Goggans, Alexander City, Ala., read a paper entitled, "Some Remarks on Appendicitis," relating fourteen cases with thirteen recoveries and one death. He thought that we were a long way from knowing the true primary cause of appendicitis, some thought it due to interference with drainage of the tube. This occurred in most cases and accounted for the pain radiating from the solar plexus, but did not account for the appendicular inflammation. Mechanical obstruction could cause pain and fatal perforation, but foreign bodies were rarely found in diseased appendix. Fitz in 300 cases had found concretions in five per cent. Dr. Goggans had found no strictly foreign bodies. Even if found it does not show that they caused the inflammation. He thought that there was something still further to be learned about the etiology; that there is a special germ which causes the disease; that the contents of the intestine became concretions and they as well as foreign bodies became lodged in the tube on account of lessened intestinal secretions and contractility of the muscular walls of the intestine and appendix. When the attack was mild (slight rise of pulse, temperature and respiration) he waited for second attack before advising removal of appendix. If at the end of two weeks there was tenderness he advised operation. In that class of cases the mortality was not more than one per cent. The more severe cases demand immediate operation, unless death is already inevitable from sepsis. Some cases might have the operation under local anesthesia more safely.

Dr. H. Horace Grant looked upon the discussion as not merely one of methods, but one that involved the security of human life, too important to dispute about contributory details; definite data must be obtained. If ever the electric rays are so perfected as to show the picture of what lies hidden beneath the tissues in cases of inflamed appendix, the question of when to operate will vex us no longer. Until then some common agreement will greatly help. Other things being equal—*i. e.*, a safe place to operate and a competent operator—the mortality will be less if all cases are cut; but these desirable conditions are only occasionally present. He suggested three rules for guidance: 1. In all cases of fulminating appendicitis, difficult to picture but easily recognized, the operation is imperative at the earliest moment before general peritonitis develops. 2. In mild or moderate cases in which operation is declined or hesitated about, or where conveniences for safety are not at hand, we are justified in waiting thirty-six or forty-eight hours to watch the course of the disease. It is to be understood that while such a course is not always safe, even when it appears to be, yet the danger does not justify an imperative demand nor an incompetent operator. 3. In all cases of moderate severity in which distinct, genuine improvement is not manifest after forty-eight hours the operation should be demanded.

Dr. F. X. Dercum, Philadelphia, Pa., read a paper on "The Pathology of Functional Diseases as a Guide to Treatment." He described the nerve cells and related observations on cell activity in lower forms of life. These show movement of nerve cells and that activity causes the cells to shrink. Alcoholism causes serious nutritional changes in cells, especially that the fine prolongations were corroded, swollen and destroyed. There may be changes in cells, destruction of same, and changes in the prolongations. Symptoms: fatigue, irritability in keeping with pathology, loss of cell substance; may be due to poisons in blood. He related experiments with frog. This is neurasthenia simplex, not that form found with organic diseases in pelvis, which is neurasthenia symptomica. The indications are: first, rest and food; second, removal of waste products. In hysteria, hypnotism should not be applied; may substitute worse condition. In insanity, rest, food, elimination of toxic agents. Autointoxication may come from: first, excess of normal constituents; second, due to diseased organs; third, poisons from without.

Dr. F. B. Sloan, Cowan, Tenn., illustrated "The Application of the Plaster Jacket and Dressings" on a subject on a frame by which the patient is to be suspended in a hammock while the jacket is applied. The patient was placed in hammock, which was supported by roller around body and around top of frame, and plaster jacket over hammock.

Dr. John A. Larrabee, Louisville, Ky., read a paper on "Some Points in the Treatment of Typhoid Fever," in which he claimed that, as we are aborting other microbic diseases, it is not unreasonable to attempt to abort typhoid. He thought that the products of the typhoid fever bacillus were far less injurious than the heterogeneous microbes and ptomaines formed in the intestinal tract by reason of improper diet and from poisonous gases, the product of putrefactive changes, all of which are in our power to prevent. Intestinal antisepsis is the *sine qua non* of treatment.

Avoid narcotics, lest the already cloudy typhoid cerebrum should forget to preside over the function of life. May occasionally be used with restlessness, jactation and a dilated pupil, especially accompanying hemorrhages. Physical examination should be made every visit. Tympanites should not be allowed above the spinous process of ilium. For this use—

℞ Ol. terebinth........................................dr. j.
Rochelle salts ........................................oz. j.
Glycerine ........................................oz. iv.

M. Inject one-half in pint of water and remainder if not relieved in two hours.

He indorsed the principle on which Dr. Woodbridge bases his treatment of intestinal antisepsis. The annoyance of the fifteen-minutes dosage advocated by Woodbridge, he thought deleterious. He advocated specific treatment—*i. e.*, all intestinal antiseptics which are innocuous.

Hyperpyrexia is important, but must be overcome by means not prejudicial to patient. When temperature persists at 104, thirty drops externally to small area of abdomen. Never use antipyrin or acetanilid. If pulse and temperature rate greatly disturbed use small doses of strychnia.

It is a question whether the enforced feeding of the present day is not as deleterious as the venesection almost to exsanguination of former days. Complete anorexia helps to make early diagnosis. Bouillon, Axtell's capsules, buttermilk, Mellin's food, with milk and plenty of sterilized water are quite sufficient to choose from, and never more than a gill at a time. He protested against the use of alcoholic stimulants. Had never seen any benefit from their use which could not have been obtained from tea or coffee. Digitalis has no place in typhoid. Strychnia is the one heart stimulant to be used. Should not wait for urgent and alarming symptoms before giving small doses. Whenever the pulse and temperature are disturbed in the relation of ten to one, strychnia should be used. Heat is a powerful stimulant to heart, and hot water bag or Geyser's hot appliance should be at hand for emergencies. Antiseptic treatment of value, not from destruction of bacilli, but from prevention of formation of ptomaines and poisonous gases, which are main factors in keeping up disease. Typhoid is a swallowed poison, hence importance of pure water. Nothing equals chlorinated lime for disinfecting the dejecta. It is as criminal to deposite the non-disinfected stools in a vault or cesspool as to put Paris green in a neighbor's well.

Dr. Wm. R. Blue, Louisville, Ky., read a paper, "The Cystoscope in the Diagnosis of Bladder and Kidney Diseases," with presentation of the Leiter-Brenner Cystoscope, which he preferred. He related cases in which it had been used for foreign bodies, stone, tuberculosis, etc. The cystoscope is indicated in obscure urinary troubles and to aid in catheterizing the ureters.

Dr. W. Frank Glenn said that value of cystoscope and endoscope could be appreciated only by those who used them. Stone can be diagnosed where not found with sound. He related a case where stone was found *post mortem* which could not be found with sound. In all obscure cases cystoscope should be used. Extremely difficult to catheterize the male ureter.

The officers elected for the ensuing year were as follows: President, Dr. J. A. Goggans, Alexander City, Ala.; Vice-Presidents, Dr. Andrew Boyd, Scottsboro, Ala.; Dr. G. W. Drake, Chattanooga; Dr. C. M. Drake, Atlanta; Secretary, Dr. Frank Trester Smith, Chattanooga; Treasurer, Dr. George R. West, Chattanooga. Dr. George S. Brown of Birmingham was elected chairman of the Committee on Arrangements. The next place of meeting: Birmingham, Ala.

